# The new trauma steering system in Stockholm – has it made a difference?

**DOI:** 10.1186/1757-7241-21-S1-S1

**Published:** 2013-05-28

**Authors:** R Rubenson, KH Wahlin, M Castrén

**Affiliations:** 1 Karolinska Institute, Stockholm, Sweden

## Introduction

A new trauma steering system was implemented in Stockholm in 2007. Prior to the change all trauma patients were transported to the nearest hospital. The new directive stipulates trauma patients to be transported to the Karolinska University Hospital, a level I trauma centre. International studies have shown positive effects after the implementation of trauma systems, but there is still no evaluation of the new system in Stockholm.

## Aims

To evaluate the impact of the new steering system on transport times, care times and compliance to the new directive.

## Material and methods

A retrospective registry study, comparing year 2006 and 2008. The study population consisted of trauma patients admitted to Karolinska University Hospital and Södersjukhuset), (N=1163). Inclusion criteria were transport by ambulance or helicopter, Injury Severity Score (ISS) > 9 and age > 15 years.

Outcomes were transport time, time on scene, time at the Intensive Care Unit, (ICU) and total hospital stay. One subgroup analysis was made for patients with ISS > 15.

## Results

A larger number of patients were steered to the trauma center in accordance with the directive, but 135 trauma patients were transported to Södersjukhuset still in 2008. The number of patients in need of secondary transfer from Södersjukhuset to the trauma center was reduced from 31 to 11 between the years. The study showed shorter times spent in the ICU at Södersjukhuset, median 0.81 vs. 0.31 days (p-value 0.002), but no significant effects on prehospital times measured.

## Conclusions

There were no impact on prehospital transport times after implementation of the new steering system but the amount of interhospital transfers decreased. The compliance to the directive was suboptimal which needs further attention.

## References

[B1] Celso B Tepas J Langland-Orban B Pracht E Papa L Lottenberg L A systematic review and meta-analysis comparing outcome of severely injured patients treated in trauma centers following the establishment of trauma systems J Trauma 2006 60 2 371 8 10.1097/01.ta.0000197916.99629.eb 16508498

[B2] Kristiansen T Soreide K Ringdal KG Rehn M Kruger AJ Reite A Trauma systems and early management of severe injuries in Scandinavia: review of the current state Injury 2010 41 5 444 52 10.1016/j.injury.2009.05.027 19540486

[B3] Garwe T Cowan LD Neas B Cathey T Danford BC Greenawalt P Survival benefit of transfer to tertiary trauma centers for major trauma patients initially presenting to nontertiary trauma centers Acad Emerg Med 2010 17 11 1223 32 10.1111/j.1553-2712.2010.00918.x 21175521

